# Development and Validation of Liquid Chromatographic Method for Estimation of Naringin in Nanoformulation

**DOI:** 10.1155/2014/864901

**Published:** 2014-03-06

**Authors:** Kranti P. Musmade, M. Trilok, Swapnil J. Dengale, Krishnamurthy Bhat, M. S. Reddy, Prashant B. Musmade, N. Udupa

**Affiliations:** Department of Pharmaceutical Quality Assurance, Manipal College of Pharmaceutical Sciences, Manipal University, Manipal 576104, India

## Abstract

A simple, precise, accurate, rapid, and sensitive reverse phase high performance liquid chromatography (RP-HPLC) method with UV detection has been developed and validated for quantification of naringin (NAR) in novel pharmaceutical formulation. NAR is a polyphenolic flavonoid present in most of the citrus plants having variety of pharmacological activities. Method optimization was carried out by considering the various parameters such as effect of pH and column. The analyte was separated by employing a C_18_ (250.0 × 4.6 mm, 5 *μ*m) column at ambient temperature in isocratic conditions using phosphate buffer pH 3.5: acetonitrile (75 : 25% v/v) as mobile phase pumped at a flow rate of 1.0 mL/min. UV detection was carried out at 282 nm. The developed method was validated according to ICH guidelines Q2(R1). The method was found to be precise and accurate on statistical evaluation with a linearity range of 0.1 to 20.0 *μ*g/mL for NAR. The intra- and interday precision studies showed good reproducibility with coefficients of variation (CV) less than 1.0%. The mean recovery of NAR was found to be 99.33 ± 0.16%. The proposed method was found to be highly accurate, sensitive, and robust. The proposed liquid chromatographic method was successfully employed for the routine analysis of said compound in developed novel nanopharmaceuticals. The presence of excipients did not show any interference on the determination of NAR, indicating method specificity.

## 1. Introduction

The alternative system of medicine is gaining importance recently. The aim of using plant isolates such as flavonoids, terpenoids, alkaloids, resins, and proanthocyanidins is increasing nowadays. Flavonoids, commonly used in human diet, are a group of naturally occurring polyphenolic compounds that are abundant in many vascular plants. These bioactive compounds have gained interest recently because of their broad pharmacological activities including antioxidant, blood lipid and cholesterol lowering, anti-inflammatory, anticarcinogenic, antiulcer, and antimicrobial, superoxide scavenging actions. Recent attention towards these substances has been stimulated by their potential health benefits [1–7].

Pure herbal constituents are used these days as components in targeted drug delivery system for achieving various pharmaceutical benefits. These flavonoids have prominent bioactivity but the major problems associated with them are limited solubility and permeability accounting for their poor bioavailability [8]. To improve the bioavailability of these components, the use of novel pharmaceutical technology is important. The various novel formulations such as nanosuspensions, liposomes, phytosomes, transfersomes and ethosomes can be prepared for bioactives to demonstrate effective and enhanced drug targeting. The utilization of the novel drug delivery system facilitated the drug administration of phytoconstituents more incisively than conventional drugs. Novel drug delivery system is advantageous due to improvement of bioavailability of the drug by delivering the drug at predetermined rate at the site of action which may reduce the side effects [9].

Naringin (NAR), a citrus flavonoid, is available as one of the most promising compound in human diet.

Naringin is also proved to enhance the bioavailability of drugs such as paclitaxel and diltiazem when administered concomitantly [10, 11]. Recently, applications of polyphenols have been of great interest in the area of functional foods, nutraceutical, and pharmaceuticals. Number of chromatographic methods are available for the estimation of naringin in grape and citrus fruits [12, 13]. There were reports of quantification of naringin in Chinese medicine and Citrus herbs using liquid chromatography [14–16]. Simultaneous diastereomeric separation of naringin and neohesperidin by normal-phase HPLC in commercial samples and herbal medicines was reported [17]. Determination of naringin in orange juice by LC with postcolumn derivatization was reported [18]. There are some LC-MS methods developed for estimation of naringin in citrus juice [19, 20]. Some of these methods are not validated, some are time-consuming, and some require expensive instruments or laborious extraction techniques and most of the methods used complex mobile phase.

For developing new dosage form of these flavonoids there is need of an analytical methodology to quantify in-process quality control samples, dissolution study samples, and final formulations.

The ambit of the present work was on development of isocratic high performance liquid chromatographic method for estimation of NAR in developed nanoformulations. The developed method was used for quantification of the NAR for assay, content uniformity, solubility studies, and drug release study of the developed formulations. The developed method is simple, precise, accurate, and rapid. The developed method was validated according to the International Conference on Harmonization (ICH) Q2(R1) guideline [21].

## 2. Material and Methods

### 2.1. Chemicals and Reagents

The working standard, Naringin (99.18%) was procured from Sigma-Aldrich, USA. Potassium dihydrogen phosphate and orthophosphoric acid AR grade were purchased from Merck India Pvt. Ltd (Mumbai, Maharashtra, India). HPLC solvents like acetonitrile and methanol were procured from Ranchem Ltd, (Mumbai, Maharashtra, India). Ultrapure water was produced in the laboratory from Siemens water purification system. All other chemicals used were of analytical grade.

### 2.2. Apparatus and Chromatographic Conditions

The analysis was carried out on Shimadzu prominence HPLC separation module with configuration of LC-20AD binary pumps along with DGU-20A5 degasser unit, SPD-20A dual *λ* UV detector, SPD-M10Avp Photodiode array detector, SIL-20AC HT autosampler, and CTO-10AS vp column oven. System control, data acquisition, and processing were performed with LC Solutions chromatography software (Version 1.24 SP1). Standard substances were weighed on Sartorius CP 225D analytical balance. A glass vacuum-filtration apparatus (Alltech Associates) was employed for the filtration of buffer solution using 0.22 *μ*m filter obtained from Pall Pvt. Ltd (Bangalore, India). Degassing of the mobile phase was performed by ultrasonication in Oscar Micro clean-103 Ultrasonic bath.

GraceSmart RP C_18_ (250.0 × 4.6 mm, 5 *μ*m) column was used as a stationary phase. The isocratic mobile phase used was acetonitrile and potassium phosphate buffer (25.0 mM; pH 3.5 ± 0.1, containing 0.2% triethylamine; pH adjusted using dilute orthophosphoric acid) in the ratio of 25 : 75% v/v. The mobile phase was pumped at a flow rate of 1.0 mL/min. The column was maintained at a temperature of 25°C and the eluent was monitored at 282.0 nm. All solutions were injected using acetonitrile: ultrapure water (1 : 1) as diluent. The injection volume was 20 *μ*L with a total run time of 10.0 min.

### 2.3. Method Optimization

#### 2.3.1. Effect of pH

For the present study, separation was carried out on GraceSmart RP C_18_ (250.0 × 4.6 mm, 5 *μ*m) column. The buffer in mobile phase was optimized using 25.0 mM of potassium dihydrogen phosphate containing 0.2% triethylamine (pH adjusted to 3.5 or 7.0) and ammonium acetate buffer (pH 5.0). The analysis of NAR was carried out using 75% of the buffer in acetonitrile. The chromatographic factors such as capacity factor, theoretical plates, and 10% asymmetry factor were calculated using chromatographic data software LC Solutions 1.24 SP1.

#### 2.3.2. Effect of Stationary Phases

The standard solution of NAR with concentration of 10.0 *μ*g/mL was injected on various columns such as Water Symmetry C_18_ (250.0 × 4.6 mm, 5 *μ*m), Merck Hibar C_18_ (250.0 × 4.6 mm, 5 *μ*m), Kromasil C_18_ (250.0 × 4.6 mm, 5 *μ*m), and GraceSmart RP C_18_ (250.0 × 4.6 mm, 5 *μ*m) and chromatograms were recorded. The chromatographic factors such asymmetry (10%), capacity factor, and theoretical plates of NAR were monitored.

#### 2.3.3. Effect of Peak Modifier

The standard solution of 10.0 *μ*g/mL of NAR was used for evaluation of the effect of peak modifier in the mobile phase. The chromatogram was recorded with and without peak modifier, that is, 0.2% triethylamine in phosphate buffer (25 mM of buffer adjusted to pH 3.5 ± 0.1 with dilute orthophosphoric acid). The chromatographic factors such as capacity factor, theoretical plate, and 10% asymmetry factor were monitored.

### 2.4. Method Validation

The present method was developed and validated in accordance with the ICH Q2(R1) guideline to fulfil the criteria set by regulatory guidelines

#### 2.4.1. Specificity

To determine the specificity of the developed method in presence of excipients, NAR was spiked with the excipients used for the preparation of nanoformulation. The drug was extracted from these excipients by sonication in methanol for 5 min followed by filtration through 0.22 *μ*m syringe filter. The filtered solution was diluted to obtain solution of 10.0 *μ*g/mL of NAR and injected into the HPLC system. A placebo formulation was processed separately as mentioned above and injected into the HPLC system followed by the standard NAR solution (10.0 *μ*g/mL). The developed formulation was also analysed by HPLC-PDA detector to check the peak integrity as peak purity. The chromatograms of placebo formulation and standard NAR were compared to monitor interferences from excipients at the retention time of NAR.

#### 2.4.2. Linearity and Range

The linearity plot was constructed for NAR in the range of 0.1 to 20.0 *μ*g/mL. The primary stock solution of 1.0 mg/mL of NAR was prepared in methanol. From the primary stock solution, secondary stock solution was prepared to get the concentration of 100.0 *μ*g/mL of NAR. Appropriate dilution of the primary and secondary stock solutions was carried in diluent to get concentrations of 0.1, 0.2, 0.5, 1.0, 2.0, 6.0, 10.0, and 20.0 *μ*g/mL for NAR. The calibration curve was plotted as concentration of the respective drug solutions versus the peak area at each level for five days. The coefficient of determination (*r* ^2^), slope, and intercept values were determined and statistically evaluated.

#### 2.4.3. Precision

Precision is the closeness of the analytical results achieved from a set of replicate measurements under the conditions of the method. Precision reflects the random errors which occur in a method [22].

Precision is usually measured as the coefficient of variation or relative standard deviation of analytical results acquired from independently prepared quality control standards.

The precision of the method was determined at three different levels covering entire range of linearity. Three different standards were prepared which contained 0.75, 5.0, and 15.0 *μ*g/mL of NAR.

The intraday precision was evaluated by analysing six sample solutions (*n* = 6) at each level in two different sets in a day. Similarly, the interday precision was evaluated in three consecutive days (*n* = 18). The NAR concentrations were determined and the relative standard deviations (RSD) were calculated.

#### 2.4.4. Accuracy

In order to evaluate the accuracy of the proposed method, a recovery test was performed by adding a known amount of standard solutions to the placebo formulation before extraction, followed by analysis using the proposed method.

The recovery studies were done for three different levels at 80%, 100%, and 120% of assay concentration using standard spiking method.

The placebo formulation was spiked with 8.0, 10.0, and 12.0 *μ*g/mL of standard NAR. The prepared samples were analysed using proposed chromatographic conditions. The amount recovered was using the linearity curve.

The percentage of recovery was calculated according to the following formula:

(1) $$\begin{array}{c} \text{Recovery}\left(\%\right)=\frac{\left(\text{Amount}\;\text{found}\;\text{in}\;\text{spiked}\;\text{placebo}\;\text{sample}\right)}{\text{spiked}\;\text{amount}}\times 100. \end{array}$$

#### 2.4.5. Limit of Detection (LOD) and Limit of Quantitation (LOQ)

LOD is the ability of analytical method to detect the lowest concentration of the analyte. LOQ is lowest concentration of the analyte which can be quantitatively determined with acceptable precision and accuracy.

The LOD and LOQ were determined by the following equation according to ICH guideline:

 LOD = 3.3 × *σ*/*s*,
 LOQ = 10 × *σ*/*s*,
 
*σ*—Standard deviation of blank response,
 
*s*—Slope of regression equation.

#### 2.4.6. Robustness

The importance of establishing robustness of the developed method by the comparison of series of system suitability parameters obtained by employing deliberate changes is to ensure the integrity of analytical procedure whenever it is used. In the present study, the assay concentration 10.0 *μ*g/mL of NAR was used for the determination of the robustness of the method. The following parameters were considered for the robustness:

(i) effect of pH of buffer in mobile phase (±0.2),
(ii) effect of mobile phase composition (±2%),
(iii) effect of wave length (±2 nm),
(iv) effect of flow rate (±10%).

#### 2.4.7. Solution Stability

NAR belongs to the category of flavonoids which calls for checking its stability. It is necessary to carry out the stability of bioactive compounds given their property of undergoing various forms of degradation. The solution stability of NAR was carried out at bench top, refrigerated condition and in autosampler. Stock solution of 100.0 *μ*g/mL of NAR was prepared in methanol. The solution was diluted using diluent to get the concentration of 10.0 *μ*g/mL of NAR and subjected to bench top and autosampler stability conditions. The stock solution was subjected to refrigerated stability condition and monitored for 15 days as long-term stability. The samples after being subjecting to various stability conditions were injected into the HPLC system. The concentration was determined at predetermined time interval using freshly prepared calibration curve.

#### 2.4.8. System Suitability

The purpose of the system suitability test is to ensure that the complete testing system (including instrument, reagents, columns, and analysts) is suitable for the intended application.

System suitability testing is an integral part of liquid chromatographic methods which is used to verify the reproducibility of the chromatographic system for the analysis to be done. The tests are based on the concept that the equipment, electronics, analytical operations, and samples to be analysed constitute an integral system that can be evaluated as such.

In the current US-FDA guidelines on “Validation of chromatographic methods,” the following acceptance limits are proposed as initial criteria (Table 1). For the present study, 10.0 *μ*g/mL of NAR was injected six times to record the system suitability parameters.

## 3. Application of Method

Developed method was applied for quantification of the NAR in developed nanosuspension prepared using Lutrol F68 (stabilizer) by cavi-precipitation method. Various formulations were prepared by altering drug and stabilizer ratio. The nanosuspensions were lyophilized using mannitol as cryoprotectant and used further for characterization. 10 mg equivalent quantity of lyophilized formulation was weighed and transferred to 10 mL volumetric flask. 3 mL of methanol was added to the flask and sonicated for 10 min. The volume was made up to the mark by using methanol. The solution was filtered through 0.22 *μ*m syringe filter. The filtrate was further diluted to get the concentration equivalent to 10.0 *μ*g/mL in diluent. The resulting solution was analysed by proposed HPLC method.

## 4. Results and Discussion

The objective of the present study was to develop and validate liquid chromatographic method for estimation of NAR in the developed formulations.

**Figure 1:**
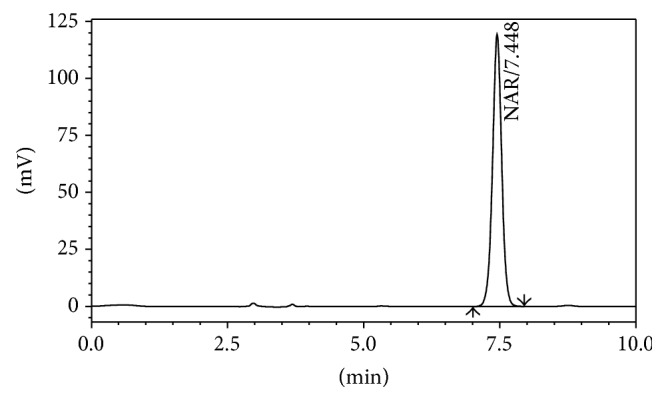
Representative chromatogram of standard NAR.

The proposed HPLC method is simple with less time consumption and requires fewer reagents. This method could be used in quality control test and estimation of the release study samples in pharmaceutical industries. The retention time of NAR was found to be 7.46 ± 0.5 min (Figure 1).

The developed method was validated as per the ICH Q2(R1) guideline to check the reliability of the method.

### 4.1. Effect of pH

**Figure 2:**
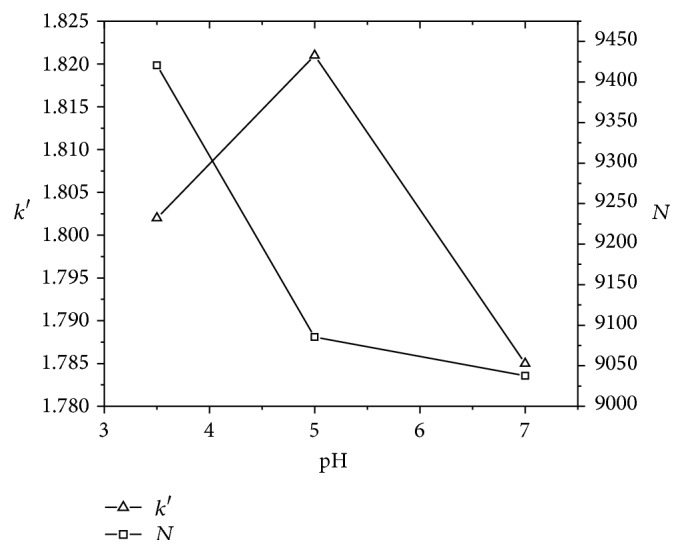
Effect of pH of capacity factor and theoretical plate of standard NAR.

By attempting the various pH of the mobile phase (pH 3.5, 5.0, and 7.0), it was observed that the retention time for NAR was found to be around 7.4 min in all the above mentioned pH and there was no significant effect of pH on the retention time. The peak shape was found to be good in pH 3.5 compared to pH 5.0 and 7.0. Hence, for the present study pH 3.5 was selected having the advantage of offering very good buffering capacity and maintaining NAR in unionized state. The effect of pH on capacity factor and theoretical plates of NAR is shown in Figure 2. The capacity factor was found to be stationed around 1.8 and with highest theoretical plate count at pH 3.5 with desired run time. The chromatographic parameters of NAR standard at various pH were tabulated in Table 4.

### 4.2. Effect of Stationary Phases

The retention time of NAR on waters Symmetry C_18_ (250 × 4.6 mm, 5 *μ*m) column was found to be less compared to Merck Hibar C_18_ (250 × 4.6 mm, 5 *μ*m), Kromasil C_18_ (250 × 4.6 mm, 5 *μ*m), and GraceSmart RP C_18_ (250 × 4.6 mm, 5 *μ*m) columns. The peak was broader and the column efficiency was less in case of Kromasil and Merck Hibar column leaving them unconsidered for the present study.

**Figure 3:**
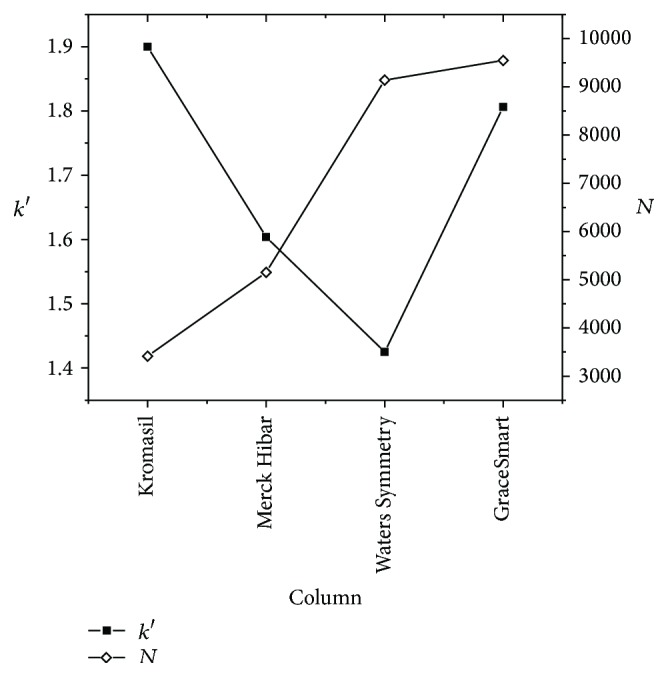
Effect of column on capacity factor and theoretical plate on standard NAR.

There was no significant change in the retention time of the NAR in all the above columns. The GraceSmart RP C_18_ (250 × 4.6 mm, 5 *μ*m) column showed good peak shape, less tailing, and acceptable retention time with better column efficiency compared to all other columns. Hence, in the present study GraceSmart RP C_18_ (250.0 × 4.6 mm, 5 *μ*m) was selected as the column for the analysis. The effect of column on chromatographic factors mainly capacity factor and theoretical plates was represented in Figure 3. The highest capacity factor that is 1.8 with highest theoretical plates count was observed with GraceSmart RP C_18_ (250.0 × 4.6 mm, 5 *μ*m). The results for system suitability parameters were tabulated in Table 4.

### 4.3. Effect of Peak Modifier

It was observed that, the 10% asymmetry factor was found to be 1.287 without TEA with low theoretical plate count. In presence of TEA the tailing factor (10%) was 1.045 with better column efficiency. The improvement of column efficiency with peak shape may be due to capping of the acidic functional groups of stationary phase resulting in the reduction of interaction of hydroxyl functional group of NAR releasing the drug quicker. Considering the optimal results, 0.2% triethylamine was used as peak modifier in the mobile phase. Results of chromatographic parameters were tabulated in Table 4.

### 4.4. Validation of the Method

#### 4.4.1. Specificity

The present method was found to be highly specific as there was no interference at retention time of the NAR from the placebo formulation.

**Figure 4:**
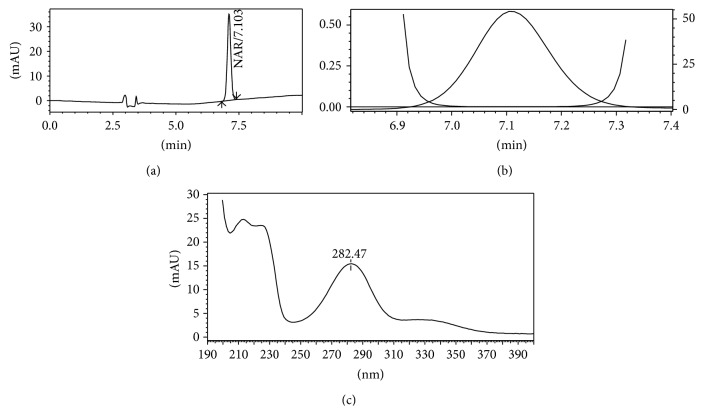
Representative chromatogram of naringin nanoformulation sample (a), and peak purity graph (b), and PDA spectrum (c).

The specificity of the method was investigated by conducting a photodiode-array analysis to investigate the integrity of the drug peaks and to clarify the purity of the peaks. The total peak purity was monitored for NAR. The peak purity was found to be greater than the peak purity threshold which indicated that the peak of NAR was pure and there was no interference from excipients. In the chromatograms of the formulations, some additional peaks were observed which may be due to presence of excipients in the formulations. The chromatogram, spectrum with peak purity chart is shown in Figure 4.

#### 4.4.2. Linearity

The response for the detector was determined to be linear over the range of 0.1 to 20.0 *μ*g/mL for NAR. The calibration curve was plotted as concentration of the drug versus the response (area of the drug peak) at each level. The proposed method was evaluated by its coefficient of determination and intercept value calculated in the statistical study. They were represented by the linear regression equation as *y* = 27494*x* + 32335 and “*r* ^2^” value = 0.99995. For all the calibration curves the coefficient of determination was within the limit of 0.9999585 ≤ *r* ^2^ ≥ 0.9999960.

Slopes and intercepts were obtained by using regression equation (*y* = *mx* + *c*) and least square treatment of the results were used to confirm linearity of the developed method.

#### 4.4.3. Precision

The % CV of interday and intraday precision obtained was less than 1% for NAR. The precision was carried out at three different concentrations of NAR over the linearity. The intraday and interday precision of NAR was in the range of 0.188–0.291 and 0.264–0.891, respectively (Table 2). From the data obtained, the developed HPLC method was found to be highly precise.

#### 4.4.4. Accuracy

The assay concentration for NAR was considered to be 10.0 *μ*g/mL. The recovery was calculated using placebo spiking method at three levels of 80–120% of the assay concentration. The mean recovery for the NAR was 99.33 ± 0.16%. The recovery data for the NAR is shown in Table 3.

#### 4.4.5. Quantification and Detection Limit

The LOD and LOQ of NAR were found to be 17.0 ng/mL and 50.0 ng/mL, respectively.

#### 4.4.6. Robustness

Method robustness was checked for NAR estimation after employing the variables to the optimised method. The data generated from the proposed method had significantly demonstrated that the HPLC method developed is robust. Results of the robustness parameter were tabulated in Table 5. It was observed that the % RSD for all the variables parameters was <1%, indicating the method is robust.

#### 4.4.7. Solution Stability

While determining the solution stability of NAR, it was observed that the methanolic solution of NAR is stable at bench top and autosampler for 12 h and 24 h, respectively. Also NAR was stable at refrigerated condition for 15 days.

## 5. Application of the Method

The proposed method was utilized for the estimation of NAR in developed formulations. The nanoformulation assay was found in the range of 60–98% for different batches. The present method was also utilized for* in vitro* release of the developed formulations indicating the suitability of the method.

## 6. Conclusion

The proposed HPLC method for estimation of the naringin in developed nanoformulations was applied successfully. Specificity oriented method development has given a wide edge to this method for the estimation of naringin in many other formulations. Utilizing an isocratic mobile phase with commonly used column is very easy to perform to give acceptable and reproducible results. The validation of proposed method was carried out as per ICH guideline and performance data for all the parameters tested is acceptable. LOD and LOQ, established by this method, are lesser than previously described methods. The method is found to be linear in the specified range, precise, and robust. Accuracy of the method is also established for the formulation. Hence, the proposed method is rapid, simple, and can be applied to quality control analyses of formulated product.

## Figures and Tables

**Table 1:** System suitability parameters.

Parameter	Limit	Observed value
Capacity factor	*k*′ > 2	1.804 ± 0.002
Injection precision	RSD < 1% for *n* > 6	0.264
Tailing factor	*T* < 2	1.04 ± 0.02
Theoretical plates/meter	*N* > 2000	9646.5 ± 154.3

**Table 2:** The precision data of NAR by the proposed HPLC method.

Concentration (*μ*g/mL)	% RSD	
	Intraday (*n* = 12)	Interday (*n* = 18)
0.75	0.291	0.891
5.0	0.188	0.784
15.0	0.278	0.264

**Table 3:** The accuracy data of NAR by the proposed HPLC method.

Spiked level	Amount added	Amount recovered	% recovery	Mean % recovery
80%	7.86	7.79	99.11	99.19 ± 0.15
	7.83	7.76	99.11	
	7.83	7.78	99.36	

100%	10.05	10.01	99.60	99.50 ± 0.10
	10.05	9.99	99.40	
	10.06	10.01	99.50	

120%	12.04	12.07	100.25	99.30 ± 0.89
	12.000	11.817	98.48	
	11.97	11.87	99.16	

% mean recovery				99.33 ± 0.16

**Table 4:** System suitability parameter.

Parameters	Mobile phase pH			Column				Peak modifier	
	3.5	5.0	7.0	Kromasil	Merck Hibar	Waters Symmetry	GraceSmart	Without TEA	With 0.2% v/v TEA
*R*_*t*_	7.467	7.493	7.396	7.702	6.917	6.442	7.452	7.443	7.484
*T*	1.054	1.048	1.052	0.936	1.242	1.127	1.0365	1.287	1.045
*k*′	1.802	1.821	1.785	1.900	1.604	1.425	1.806	1.804	1.803
*N*	9420.4	9085.4	9037.6	3415.11	5152.9	9139.41	9546.5	7687.4	9434.5

*R*_*t*_: Peak retention time, *T*: tailing factor, *k*′: capacity factor and *N*: theoretical plates.

**Table 5:** Robustness parameter of the proposed method.

Parameters	% RSD of area of NAR
Mobile phase pH	
3.3	0.113
3.7	0.287
Mobile phase composition (acetonitrile : buffer) (% v/v)	
27 : 73	0.252
23 : 77	0.166
UV detector wavelength (nm)	
280	0.107
284	0.102
Flow rate (mL/min)	
0.9	0.097
1.1	0.363
